# Evaluation of Bone Mineral Metabolism in Pre-Dialysis Chronic Kidney Disease: Quantitative Computed Tomography vs. Dual-Energy Absorptiometry and Correlation with Bone Turnover Markers

**DOI:** 10.3390/medicina61010152

**Published:** 2025-01-17

**Authors:** Aydan Mutis Alan, Zehra Sezer, Ahmet Oz, Cebrail Karaca, Mevlüt Tamer Dinçer, Ahmet Murt, Fatma Beyza Sag, Selma Alagoz, Serdar Sahin, Mustafa Sait Gonen, Elif Güzel, Sinan Trabulus, Nurhan Seyahi

**Affiliations:** 1 Division of Nephrology, Department of Internal Medicine, Cerrahpasa Medical Faculty, Istanbul University-Cerrahpaşa, Istanbul 34098, Turkey; cebrailkaraca@gmail.com (C.K.); tamerdincer@gmail.com (M.T.D.); murtahmet@yahoo.com (A.M.); sinantrabulus@gmail.com (S.T.); nseyahi@gmail.com (N.S.); 2 Department of Histology and Embryology, Cerrahpasa Faculty of Medicine, Istanbul University-Cerrahpasa, Istanbul 34098, Turkey; zehra.sezer@iuc.edu.tr (Z.S.); beyzasag@istanbul.edu.tr (F.B.S.); elifguzelctf@yahoo.com (E.G.); 3 Department of Radiology, Cerrahpsa Faculty of Medicine, Istanbul University-Cerrahpasa, Istanbul 34098, Turkey; ozz.ahmett@yandex.com; 4 Division of Nephrology, Department of Internal Medicine, Bagcilar Training and Research Hospital, University of Health Sciences, Istanbul 34200, Turkey; alagozselma@yahoo.com; 5 Division of Endocrinology and Metabolism, Department of Internal Medicine, Cerrahpasa Medical Faculty, Istanbul University-Cerrahpasa, Istanbul 34098, Turkey; srdr_shn@hotmail.com (S.S.); gonen.sait@gmail.com (M.S.G.)

**Keywords:** dual-energy absorptiometry, quantitative computed tomography, bone turnover markers, chronic kidney disease, bone mineral density

## Abstract

*Background and Objectives*: Bone and mineral disease (BMD) is a prevalent complication of advanced chronic kidney disease (CKD). The risk of fractures can be assessed via dual-energy X-ray absorptiometry (DXA) and quantitative computed tomography (QCT). This study aims to evaluate the effectiveness of two imaging modalities in identifying bone mineral status in individuals with pre-dialysis chronic renal disease and to assess their correlation with bone turnover markers. *Materials and Methods*: This controlled cross-sectional study, conducted at a single center from 2019 to 2022, assessed two groups of individuals aged 18 to 50. The patient cohort consisted of individuals with stage 4–5 chronic kidney disease, whereas the control cohort consisted of healthy participants. The participants’ bone and mineral status was evaluated using both QCT and DXA methods. Diagnostic measurements of the lumbar spine and femoral neck, obtained using DXA and QCT, were compared. Z-scores were utilized to evaluate low bone mineral density, with low Z-scores identified in either lumbar spine or femoral neck measures being seen as indicative of low bone mineral density. *Results*: Data from 38 participants (patient group: 18; control group: 20) who underwent QCT and/or DXA were evaluated. Thirty-three subjects were assessed using both QCT and DXA (patient group: 14; control group: 19). The median age of the patient cohort was 44 (range: 22–50), whereas the median age of the control cohort was 42 (range: 27–48) (*p* = 0.72). Women constituted 33% of the patient cohort and 50% of the control cohort (*p* = 0.23). In the patient cohort, low bone mineral density was detected in four individuals (28%) through QCT, and in just two patients (14%) through DXA. Compared to DXA, QCT identified a higher number of cases of low bone mineral density in the CKD cohort; however, no statistically significant difference was observed (*p* = 0.06). In addition, our study found that TRACP5b had a strong negative correlation with the DXA L1–L4 Z-score. *Conclusions*: This study revealed that QCT may be more sensitive than DXA for detecting low bone density in pre-dialysis CKD patients. Additionally, DXA may overestimate lumbar spine BMD in this population, and the strong negative correlation between TRACP5b levels and the DXA L1–L4 Z-score highlights the potential role of biochemical markers in assessing bone status in CKD

## 1. Introduction

Chronic kidney disease (CKD) is associated with an increased risk of fractures [1]. The assessment of fracture risk currently relies on a combination of clinical factors and quantitative imaging of bone. X-ray-based instruments have been created to evaluate bone condition and predict fracture risk. Dual-energy X-ray absorptiometry (DXA) is accepted as the standard method for assessing bone mineral density (BMD) [2]. Longitudinal studies show that low areal bone mineral density (BMD), assessed via DXA, is a predictor of fractures in the chronic kidney disease (CKD) population, similar to non-uremic populations. The 2017 Kidney Disease Improving Global Outcomes (KDIGO) guideline suggests using dual-energy X-ray absorptiometry (DXA) to assess fracture risk in patients with CKD G3a-5d (Grade-2 evidence) [3].

Nevertheless, several inherent limitations associated with the use of DXA have been reported in recent studies. Initially, the T score employed to characterize osteoporosis does not reflect the absolute value of bone mineral density (BMD). Additionally, the degeneration of bones (osteophytes and/or sclerosis) and abdominal aortic calcification may lead to an overestimation of lumbar spine measurements in DXA [4].

A bone biopsy is the definitive method for identifying bone turnover and associated pathology in patients with chronic kidney disease (CKD). Histomorphometric analyses yield information about bone mineralization and volume. The biopsy procedure is currently available at select medical establishments. Furthermore, both patients and professionals are resistant to bone biopsy due to its invasive nature.

Because of the the restrictions of DXA and the difficulties faced in undertaking bone biopsies, new diagnostic techniques for CKD-MBD should be established. Quantitative computed tomography (QCT) is one of these methods. It can exclude spinal degeneration and abdominal calcification [5]. Nonetheless, the evidence concerning the efficacy of DXA and QCT in assessing the state of bones in pre-dialysis CKD patients remains unsatisfactory.

This study aims to assess bone status in the lumbar spine and femoral neck of pre-dialysis CKD patients using DXA and QCT techniques. We intended to evaluate the diagnostic efficacy of the two approaches within the same chronic kidney disease cohort and a healthy control group. Furthermore, we evaluated the relationship between bone biomarkers and both BMD and Z-score in the entire cohort.

## 2. Method

**Figure 1:**
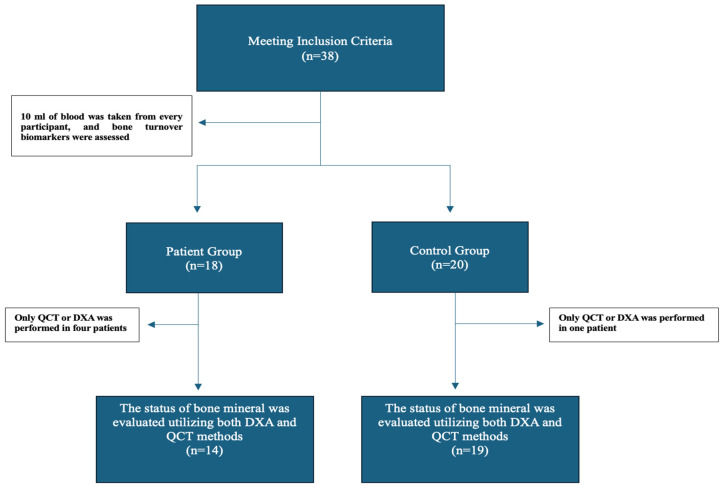
Diagram of the study design.

We conducted a cross-sectional study at a single center from 2019 to 2022. We assessed two cohorts aged 18–50 years. Premenopausal female participants were included as postmenopausal osteoporosis was considered as a confounding factor. The patient cohort comprised individuals with stage-4–5 chronic kidney disease, while the control group comprised healthy volunteers. We assessed the bone status using both QCT and DXA. Diagnostic discordance between DXA and QCT was evaluated based on lumbar spine and femoral neck Z-scores and bone mineral density (BMD). Concurrently, 10 mL of blood was taken from every participant, and bone turnover biomarkers were assessed. Patients under the age of 18 years, those receiving renal replacement therapy (hemodialysis, peritoneal dialysis, or renal transplantation), and those using bisphosphonates, calcitonin, hormone therapy, and oral contraceptives were excluded. The estimated glomerular filtration rate (eGFR) was calculated using the Chronic Kidney Disease Epidemiology Collaboration (CKD-EPI) formula [6]. The study design is illustrated in Figure 1.

### 2.1. Dual-Energy Absorptiometry

BMD at the femoral neck, total hip, and lumbar spine (L1–L4) was evaluated via DXA with Hologic QDR Apex 4500 equipment (Hologic Inc., Bedford, MA, USA). The assessments were performed by a certified DXA technician. The World Health Organization recommends utilizing the Z-score for diagnosing osteoporosis in premenopausal women and males under 50 years of age. A Z-score of −2 standard deviations or lower indicates a bone mass that is below the expected level for chronological age [7,8].

### 2.2. Quantitative Computer Tomography

The QCT images of the lumbar spine and hip were obtained using a Revolution Evo 128-slice CT scanner (G.E. Healthcare, Milwaukee, WI, USA). QCT was evaluated by the same radiologist, who was blinded to the study’s details. The diagnostic categorization recommended by the American College of Radiology was applied to the CKD patient group as follows: a vBMD below 80 mg/cm^3^ indicated osteoporosis, a vBMD between 80–120 mg/cm^3^ indicated osteopenia, and a vBMD above 120 mg/cm^3^ was considered normal [9].

### 2.3. Biomarkers of Bone Turnover

Blood samples were centrifuged, and the resulting serum was used for the subsequent tests. The protein levels of CTX I (Cross Linked C-telopeptide of Type I Collagen; Elabscience, Houston, TX, USA; E-EL-H0835), sRANKL (Soluble Receptor Activator of Nuclear factor-kB Ligand; Elabscience; E-EL-H5558), TRACP-5b (Tartrate Resistant Acid Phosphatase 5b; Elabscience; E-EL-H1551), PINP (Procollagen I N-Terminal Propeptide; Elabscience; E-EL-H0185), OC/BGP (Osteocalcin; Elabscience; E-EL-H1343), OPG (Osteoprotegerin; Elabscience; E-EL-H1341), PTH (Parathyroid; DRG.BioCheck located in South San Fransisco, CA, USA; EIA-3645), and BAP (Bone-specific Alkaline Phosphatase; QUIDEL MicroVue EIA Kit, Thermo Fisher Scientific, Waltham, MA, USA; 8012) were analyzed using commercial ELISA kits (Thermo Fisher Scientific, Waltham, MA, USA). All procedures were performed according to the manufacturer’s instructions.

### 2.4. Statistical Analysis

Descriptive statistics were used to outline the demographic and clinical characteristics, expressed as frequencies and percentages. Continuous variables were presented as median values with interquartile ranges (IQRs). The normality of the data was tested via Kolmogorov–Smirnov or Shapiro–Wilks test, and the parameters did not show a normal distribution. Chi-square or Fisher’s exact test was performed to compare categorical variables. Mann–Whitney U was used to compare the difference in medians between two independent groups. The correlation of bone turnover biomarkers with BMD and Z-score results was investigated via Spearman’s and Pearson’s rank correlation tests. *p* ≤ 0.05 value was set as the level of significance. Statistical analyses were carried out using SPSS version 22.0 (SPSS Inc., Chicago, IL, USA).

## 3. Results

### 3.1. Baseline Characteristics

Data from a total of 38 participants (18 in the patient group and 20 in the control group) who underwent QCT and/or DXA were analyzed. The median age and body mass index values were comparable between the CKD and control groups (*p* = 0.72 and 0.14, respectively). No significant difference was observed in the sex ratio between the CKD group (67% male) and the control group (50% male) (*p* = 0.23). As expected, the eGFR was significantly lower in the CKD group, while the PTH levels were significantly higher in the CKD group. No significant difference was observed in serum calcium, phosphorus, and 25-OH vitamin D3 levels among the groups. Demographic and clinical data are presented in Table 1.

### 3.2. DXA and cQCT Outcomes

Thirty-five participants were evaluated using DXA (patient cohort: 15 participants; control cohort: 20 participants). Based on the spinal Z-score, three patients (9%) in the entire cohort exhibit low bone mineral density. Two participants in the chronic kidney disease group and one healthy participant in the control group exhibit low mineral density. There is no statistically significant difference between the two groups based on the DXA spinal Z-score (*p* = 0.38). Moreover, there was no statistically significant difference in the status of bone between the two groups based on the femoral and total neck Z-scores (*p* = NA and 0.38, respectively).

Thirty-four participants were evaluated using QCT. Based on spinal BMD and Z-score, five patients (15%) and three patients (9%) have low bone mineral density in the entire group, respectively. Three patients in the CKD group and two healthy participants in the control group exhibit low mineral density, as measured via spinal BMD. The spinal Z-score indicates low mineral density in two patients from the CKD group and one healthy individual from the control group. Moreover, there is no statistically significant difference in bone condition between the two groups concerning the femoral neck and total neck QCT Z-scores (*p* = NA and 0.25, respectively). We show the DXA and QCT outcomes in Table 1.

### 3.3. Comparison of DXA and QCT Outcomes

Bone mineral status was assessed in fourteen patients from the CKD cohort using both DXA and QCT. QCT identified low bone density in four individuals (28%), while DXA detected low bone mineral density in only two patients (14%). Compared to DXA, QCT identified low bone mineral density in a higher percentage of patients in the CKD cohort. However, due to the small sample size, statistical significance was not reached (*p* = 0.06). No significant differences were seen in the assessment of bone mineral status between QCT and DXA in the control group (16% vs. 10%, *p* = 0.1, respectively) (Table 2).

In the patient cohort, when assessed based on bone regions, QCT detected statistically significantly lower bone density in the lumbar region compared to DXA [3 (21%) vs. 2(14%), *p* = 0.03)] (Table 3).

### 3.4. Correlation Between Bone Biomarker and BMD and Z-Score in the Patient Group

In the QCT group, the median CTX-1 level was statistically significantly elevated in participants with low mineral density compared to those with normal mineral density (0.36 vs. 0.22, *p* = 0.007). The median PINP value was statistically higher in participants with low mineral density (1491 vs. 941.1, *p* = 0.02). Bone marker results are indicated in Table S1 (Supplementary).

**Figure 2:**
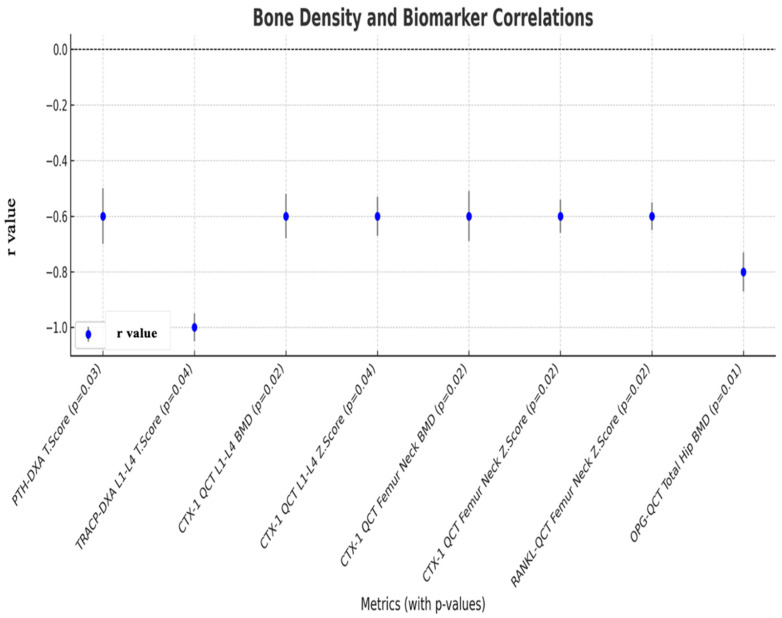
Correlation analysis results between bone mineral density parameters and bone turnover markers in the patients group.

TRACP5b (rho: −0.99 *p*: 0.04) showed a strong negative correlation with DXA L1–L4 Z-score. CTX-1 was negatively correlated with QCT L1–L4 BMD (rho: −0.59 *p*: 0.02), QCT L1–L4 Z-score (rho: 0.59, *p*: 0.04), QCT femur neck BMD (rho: −0.62, *p*: 0.02), and QCT femur neck Z-score (rho: −0.64 *p*: 0.02). A weak negative correlation was observed between RANKL (rho: −0.61, *p*: 0.02) and the QCT femoral neck Z-score. The findings of the correlation analysis are illustrated in Figure 2 (Table S2 (Supplementary Materials)).

## 4. Discussion

We conducted a cross-sectional study at a single center between 2019 and 2022. Our findings indicate that quantitative computed tomography (QCT) detected low bone density in a higher number of CKD patients compared to dual-energy X-ray absorptiometry (DXA), although the difference did not reach statistical significance due to the limited sample size. Additionally, DXA appeared to overestimate lumbar spine bone mineral density (BMD) in CKD patients prior to dialysis initiation. Furthermore, a strong negative correlation was observed between TRACP5b levels and the DXA L1–L4 Z-score, suggesting the potential value of TRACP5b as a biochemical marker in assessing bone status in this population.

Bone mineral density measurement using DXA is a valuable method for assessing bone fragility. In CKD patients, changes in bone density predominantly affect cortical bone, while trabecular bone tends to be preserved. Nevertheless, DXA is unable to distinguish between cortical and trabecular bone. The high incidence of degenerative diseases and abdominal aortic calcifications in CKD patients may cause an overestimation of lumbar spine bone mineral density when using DXA [4]. QCT can be utilized for the axial skeleton and remains unaffected by vascular calcification. It excels at distinguishing between cortical and trabecular bone, rendering it superior to DXA for identifying bone loss. Therefore, QCT offers advantages in cases where accurate differentiation between bone types and the avoidance of interference from vascular calcifications are crucial [10].

A retrospective trial by K. Kim et al., carried out in 2021, included 117 pre-dialysis chronic renal patients and 363 healthy control patients (eGFR ≥ 60 mL/min/1.73 m^2^). The bone mineral status was evaluated via DXA in the healthy control group and via both DXA and QCT in the CKD group. When utilizing the T score from the lumbar spine, osteoporosis was less common in the CKD group compared to the controls (6.8% vs. 11.0%), while normal BMD status was more prevalent in the CKD group (65.0% vs. 52.9%). On the other hand, when the femoral neck T score was assessed, osteoporosis was observed to be more prevalent in the CKD group compared to the control group (8.5% vs. 4.7%, *p*: 0.012). In the CKD cohort, QCT results demonstrated a worse status than DXA for the lumbar spine [11]. Our study indicated that QCT detected a worsened bone status than DXA for the lumbar spine in the CKD cohort, consistent with the data presented in this article. In contrast, low bone density was similar in both the CKD and control groups when assessing the lumbar region z-score and total femoral neck.

Recent studies indicate that QCT detects low BMD with greater precision than DXA, particularly in the lumbar region; however, its use remains limited. It is primarily employed in specific centers and for research purposes. Consequently, in accordance with the current KDIGO guidelines, we recommend using DXA-BMD testing to assess fracture risk in CKD G3a-G5D patients with osteoporosis risk factors and/or evidence of CKD-MBD, if the results will impact treatment decisions [3].

A bone biopsy from the iliac crest remains the gold standard for assessing bone turnover and identifying associated pathology in individuals with advanced-stage chronic kidney disease (CKD). A bone biopsy is generally conducted just inferior to the anterior superior iliac spine after double tetracycline labeling. The bone procedure is typically well-tolerated, resulting in minimal discomfort and pain. Complications associated with bone biopsy may include pain, hematoma, wound infection, and, rarely, neuropathy. Consequently, bone biopsy may be considered a safe procedure with minimal morbidity [12]. Bone biopsy assesses bone turnover, and histomorphometric analysis from the biopsy provides information on mineralization and bone volume [13]. While bone histomorphometry is the definitive method for assessing bone turnover status in patients with chronic kidney disease, the use of bone biopsy is limited by several factors. These include the invasive nature of the procedure, insufficient technical training, and a lack of centers with expertise in tissue processing and preparation [14].

At the outset of our study design, we planned to perform bone biopsies in patients with advanced renal failure. However, due to limited access to specialized laboratories capable of conducting histomorphological bone tissue analysis, we were unable to perform the biopsies.

Quantitative ultrasonography (QUS) devices are used to evaluate bone quality in individuals with chronic kidney disease (CKD). QUS employs lower-frequency waves compared to conventional soft tissue ultrasound. The skeletal regions analyzed include the distal metaphysis of the phalanx, the calcaneus, the radius, and the tibia [15]. The velocity of QUS waves traveling through bone reflects the material characteristics of the bone, such as its density, structure, and elasticity. QUS measurements have been correlated with BMD assessed via DEXA in both the phalanges and the calcaneus [16]. However, despite being non-invasive and free of radiation exposure, further studies are needed to determine its predictive value for CKD patients [17].

Microarchitecture and remodeling are interrelated; both low and high remodeling cause a loss of bone structural integrity and an elevated risk of fractures. Biomarkers of bone turnover and/or bone remodeling regulators are frequently used in clinical practice, but only some have been employed in clinical research. Although these markers are easy to measure and use, there are limitations to their application. The cut-off levels for these biomarkers are controversial, and diminished renal clearance complicates assessment, particularly in individuals with chronic renal failure. Furthermore, certain biomarkers are not exclusively indicative of metabolic processes in bone tissue; they may also be expressed in other tissues and could indicate non-primary bone disorders [18].

Certain bone turnover markers (BTMs), including osteocalcin and the C-terminal telopeptides of type I collagen (CTX), are cleared from circulation by the kidney [19,20]. Others, including bone-specific alkaline phosphatase (BSAP), procollagen type-1 N-terminal propeptide (P1NP), and tartrate-resistant acid phosphatase-5b (Trap-5b), are metabolized via non-renal pathways [21,22]. BTMs metabolized via non-renal pathways could provide more accurate assessments of low BMD risk in CKD. Praopilad S. et al., in their review entitled “Clinical Use of Bone Turnover Markers in Chronic Kidney Disease”, stated that CTX and total P1NP are the recommended markers for monitoring treatment and predicting fractures in osteoporosis patients. However, they noted that their use in CKD is limited, as CTX is cleared by the kidneys. In contrast, bone ALP and TRACP5b are not affected by renal clearance, making them reliable markers for studying renal osteodystrophy in CKD. Current literature studies investigating the relationship between BTM and fracture risk have reported inconsistent results. While some studies have found a positive association between prevalent fractures in CKD G2-5D and increased levels of PTH, bone ALP, TRACP5b, and P1NP, others have not identified such a relationship [23,24].

The cross-sectional study by Thomas L. et al. investigated the correlations between biochemical markers and bone parameters, such as areal bone mineral density (aBMD), volumetric bone mineral density (vBMD), bone size, and microstructure. Elevated bone formation markers were linked to lower aBMD (measured via DXA) and reduced vBMD with microstructural deterioration (assessed vua QCT). Specifically, osteocalcin and P1NP showed significant inverse correlations with aBMD in the femur neck, radius, and total hip, as well as with total and trabecular vBMD at the radius and tibia. Increased P1NP levels correlated with reduced cortical area and density at the tibia. Additionally, higher resorption markers, including CTX and TRAP5b, were associated with decreased aBMD and vBMD and impaired microarchitecture, especially in trabecular regions of the radius and tibia. In this study, it was also shown that the 23 patients who experienced fractures had low BMD at the femoral neck and elevated levels of Oc, PINP, and TRACP5b [25]. Similarly, we found significant negative correlations between TRACP5b and DXA L1–L4 Z-scores, as well as between CTX-1 and QCT L1–L4 Z-scores, QCT femur neck BMD, and Z-scores. A weak negative correlation was also observed between RANKL and QCT femoral neck Z-scores in our study.

**Figure 3:**
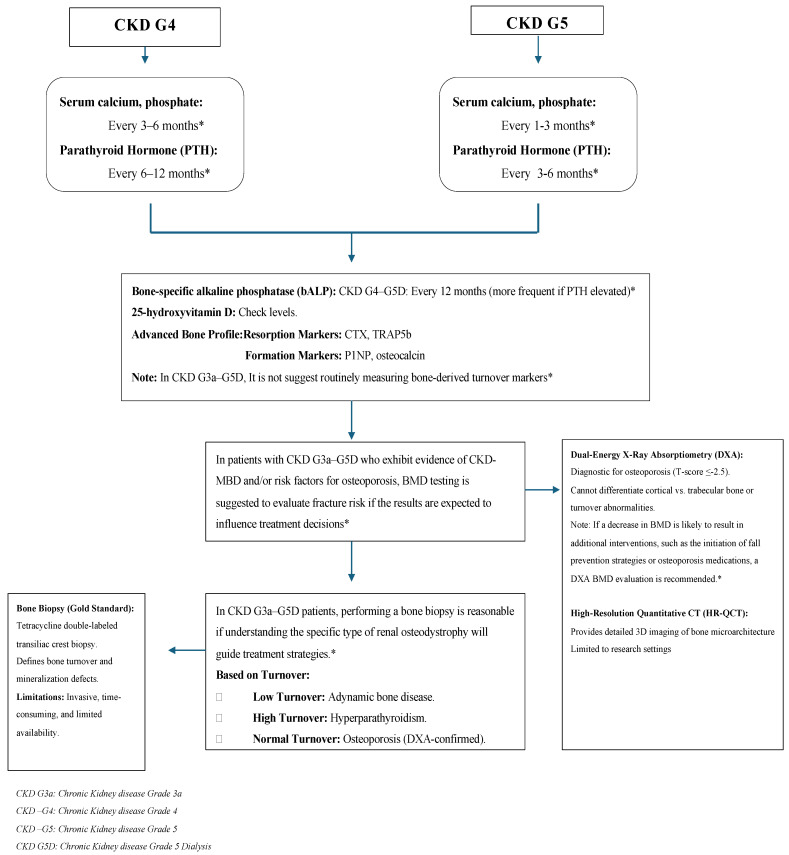
CKD BMD diagnosis and follow-up algorithm for patients with stage-4 and stage-5 CKD according to the current guidelines and the literature. * According to KDIGO 2017 guideline [3].

According to current guidelines and the literature, the CKD BMD diagnosis and follow-up algorithm for patients with stage-4 and stage-5 CKD is summarized in Figure 3.

The main limitations of our study include the relatively small population, it being a single-center study, and the cross-sectional design. Due to limited funding from our university, the budget allocated for imaging and bone turnover markers was restricted. As a result, the number of patients included in this study was limited. We could not perform a bone biopsy, which is the gold-standard procedure for diagnosing bone mineral density (BMD) status.

## 5. Conclusions

In conclusion, while the difference did not reach statistical significance due to the limited sample size, our study suggests that QCT may be a more sensitive tool for detecting low bone density in pre-dialysis chronic kidney disease patients compared to DXA. Furthermore, our findings indicate that DXA may overestimate lumbar spine BMD in this patient population. Additionally, the strong negative correlation between TRACP5b levels and the DXA L1–L4 Z-score further underscores the potential role of biochemical markers in assessing bone status in CKD patients.

## Figures and Tables

**Table 1:** Baseline demographic and clinical findings of the whole cohort. * Mann–Whitney U test. ** Student *t*-test. NA: not applicable. *** Three patients’ Z-scores were not calculated.

			Patients (*n* = 18)	Control (*n* = 20)	*p*	Total (*n* = 38)
Gender		Female	6 (33%)	10 (50%)	0.23	16 (42%)
		Male	12 (67%)	10 (50%)		22 (58%)
Age (median) (range)			44 (22–50)	42 (27–48)	0.72 *	42 (22–50)
BMI (median) (range) (kg/m^2^)			25.4 (20.7–33.2)	28.3 (21.6–34.7)	0.14 **	27.7 (20.7–34.7)
eGFR (median) (range) (mL/min/1.73 m^2^)			17.5 (6–28)	112.5 (63.7–120)	<0.0001 **	28 (6–120)
Calcium (median) (range) (mg/dL)			9.00 (7.2–9.9)	9.20 (8.1–10.2)	0.05	9.08 (7.2–10.2)
Phosphorus (median) (ange) (mg/dL)			4.5 (3.1–6.5)	4.1 (3.1–4.56)	0.08 *	4.1 (3.1–6.5)
Parathormone (median) (range) (pg/mL)			159.2 (19–630)	41.2 (23–87.3)	<0.0001 *	73.5 (19–630)
25-OH vitamin D3 (median) (range) (ng/mL)			13.8 (4.8–35)	12.0 (3–35)	0.53 *	13.7 (3–35)
DXA (*n* = 35)	Spinal Z-Score	Normal	13 (87%)	19 (95%)	0.38	32 (91%)
		Low mineral density	2 (13%)	1 (5%)		3 (9%)
	Femoral Neck Z-Score	Normal	15 (100%)	20 (100%)	NA	35 (100%)
		Low mineral density	0	0		0
	Total Neck Z-Score	Normal	15 (100%)	19 (95%)	0.38	34 (97%)
		Low mineral density	0	1 (5%)		1 (3%)
QCT *** (*n* = 34)	Spinal BMD	Normal	12 (80%)	17 (90%)	0.43	29 (85%)
		Low mineral density	3 (20%)	2 (10%)		5 (15%)
	Spinal Z-Score	Normal	13 (87%)	18 (95%)	0.41	31 (91%)
		Low mineral density	2 (13%)	1 (5%)		3 (9%)
	Femoral Neck Z-Score	Normal	15 (100%)	19 (100%)	NA	34 (100%)
		Low mineral density	0	0		0
	Total Neck Z-Score	Normal	14 (94%)	19 (100%)	0.25	33 (97%)
		Low mineral density	1 (6%)	0		1 (3%)

**Table 2:** Bone mineral density results in the whole cohort.

	Patient Group			Control Group		
	DXA (*n* = 14)	QCT (*n* = 14)	*p*	DXA (*n* = 19)	QCT (*n* = 19)	*p*
Normal bone mineral density	12 (86%)	10 (82%)	0.06 *	17 (90%)	16 (84%)	0.16 *
Low mineral density	2 (14%)	4 (28%)		2 (10%)	3 (16%)	

* Fisher’s exact test.

**Table 3:** Comparative data of QCT and DXA in the patient group.

			Patient Group		
			QCT (*n* = 14)		
			Normal	Low Mineral Density	*p*
DXA (*n* = 14)	L1–L4 (*n* = 14)	Normal	11	1	0.03 *
		Low mineral density	0	2	
	Femoral neck Z-score (*n* = 14)	Normal	14	0	NA
		Low mineral density	0	0	
	Total neck Z-score (*n* = 14)	Normal	13	1	NA
		Low mineral density	0	0	

* Fisher’s exact test.

## Data Availability

All the data supporting our findings are contained within the manuscript.

## Supplementary Materials

The following supporting information can be downloaded at: https://www.mdpi.com/article/10.3390/medicina61010152/s1, Table S1. Bone turnover marker results according to bone mineral density status via DXA and QCT in the whole population; Table S2. Correlation analysis results between bone mineral density parameters and bone turnover markers.
