# Supplementary material for: Evaluation of Bone Mineral Metabolism in Pre-Dialysis Chronic Kidney Disease: Quantitative Computed Tomography vs. Dual-Energy Absorptiometry and Correlation with Bone Turnover Markers
Source: Medicina (Kaunas). 2025 Jan 17;61(1):152. doi: 10.3390/medicina61010152 (PMC11767074; doi:10.3390/medicina61010152)
Supplement: Supplementary file 1 [file medicina-61-00152-s001.zip › medicina-3414906-supplementary.pdf]

Tablo S1. Bone turnover marker results according to bone mineral density status by DXA and QCT in the whole population

Median (range)	DXA-			QCT		
	Normal bone mineral density	Low bone mineral density	P	Normal bone mineral density	Low bone mineral density	P
BAP*	34.47 (19.3-126.6)	27.4 (13.9-33.9)	0.06	33.9 (19.3-126.6)	32.7 (13.9-82.3)	0.81
CTX1	0.25 (0.01-1.4)	0.22 (0.05-0.72)	0.87	0.22 (0.02-0.71)	0.36 (0.05-1.4)	**0.007**
PTH	646.9 (506.9-692.4)	559.8 (559.8-621.9)	0.11	641.5 (506.9-692.4)	626.8 (559.8-688.5)	0.85
sRANKL	101.4 (0.09-274.5)	157.55 (29.7-250.5)	0.27	93.16 (16.2-274.5)	147.9 (29.7-258.8)	0.05
TRACP	33.79 (10.92-53.97)	39.21 (28.06-47.07)	0.46	34.43 (10.92-53.97)	43.20 (23.61-49.82)	0.22
PINP	1004.0 (238.9-1725)	1337.5 (571.5-1491)	0.55	941.1 (238.9-1725)	1491 (571.5-1688)	**0.02**
OCBGP	33.7 (0.27-85.9)	26.03 (3.8-48.61)	0.45	33.7 (0.27-85.)	45.7 (3.8-80.0)	0.77
OPG*	1.91 (0.3-16.2)	2.42 (1.2-2.8)	0.88	1.91 (0.3-16.2)	2.79 (1.2-5.1)	0.40

** Mann-Whitney U test*

CTX I (Cross Linked C-telopeptide of Type I Collagen;), sRANKL (Soluble Receptor Activator of Nuclear factor-kB Ligand), TRACP-5b (Tartrate Resistant Acid Phosphatase 5b), PINP (Procollagen I N-Terminal Propeptide), OC/BGP (Osteocalcin), OPG (Osteoprotegerin), PTH (Parathyroid hormone), and BAP (Bone-specific Alkaline Phosphatase)

Table S2. Correlation analysis results between bone mineral density parameters and bone turnover markers

Whole group	
PINP -DXA TOTAL NECK Z SCORE	r: -0.36, p=0.042
PINP-QCT FEMUR NECK Z SCORE	r: -0.51, p=0.004
PINP-QCT TOTAL HIP BMD	r: -0.44, p=0.017
PINP-QCT TOTAL HIP Z SCORE	r: -0.44, p=0.017
RANKL-DXA FEMUR NECK Z SCORE	r: -0.36, p=0.043
RANKL-QCT FEMUR NECK Z SCORE	r: -0.44, p=0.017
RANKL-QCT FEMUR NECK BMD	r: 0.40, p=0.031
CTX-1 – DXA L1-L4 Z SKORU	r:-0.40, p=0.02
CTX-1- QCT L1-L4 Z SKORU	r:-0.43, p=0.02
CTX-1 QCT L1-L4 BMD	r:-0.41, p=0.019
**Control Group**	
PINP-DXA L1-L4 Z SCORE	r: -0.59, p=0.009
PINP-DXA L1-L4 BMD	r:-0.50, p=0.03
PINP-QCT FEMUR Z SCORE	r: -0.51, p=0.03
RANKL-QCT FEMUR NECK BMD	r: 0.72, p=0.001
**Patients Group**	
PTH-DXA TOTAL NECK Z SCORE	r:-0.61, p=0.03
TRACP-DXA L1-L4 Z SCORE	r:-0.99, p=0.04
CTX-1 – QCT L1-L4 BMD	r:-0.59, p=0.02
CTX-1 – QCT L1-L4 Z Score	r:-0.59, p=0.04
CTX-1 – QCT Femur Neck BMD	r:-0.62, p=0.02
CTX-1 – QCT Femur Neck Z Score	r:-0.64, p=0.02
RANKL – QCT FemurNeck Z Score	r:-0.61, p=0.02
OPG- QCT Total HİP BMD	r:-0.70, p=0.01

CTX I (Cross Linked C-telopeptide of Type I Collagen;), sRANKL (Soluble Receptor Activator of Nuclear factor-kB Ligand), TRACP-5b (Tartrate Resistant Acid Phosphatase 5b), PINP (Procollagen I N-Terminal Propeptide), OC/BGP (Osteocalcin), OPG (Osteoprotegerin), PTH (Parathyroid hormone), and BAP (Bone-specific Alkaline Phosphatase)
